# Adolescent with COVID-19 as the Source of an Outbreak at a 3-Week Family Gathering — Four States, June–July 2020

**DOI:** 10.15585/mmwr.mm6940e2

**Published:** 2020-10-09

**Authors:** Noah G. Schwartz, Anne C. Moorman, Anna Makaretz, Karen T. Chang, Victoria T. Chu, Christine M. Szablewski, Anna R. Yousaf, Marie M. Brown, Ailis Clyne, Amanda DellaGrotta, Jan Drobeniuc, Jacqueline Korpics, Adam Muir, Cherie Drenzek, Utpala Bandy, Hannah L. Kirking, Jacqueline E. Tate, Aron J. Hall, Tatiana M. Lanzieri, Rebekah J. Stewart

**Affiliations:** ^1^CDC COVID-19 Response Team; ^2^Epidemic Intelligence Service, CDC; ^3^Rhode Island Department of Health; ^4^Georgia Department of Public Health; ^5^Cook County Department of Public Health, Oak Forest, Illinois; ^6^Massachusetts Department of Public Health.

There is increasing evidence that children and adolescents can efficiently transmit SARS-CoV-2, the virus that causes coronavirus disease 2019 (COVID-19) (*1*–*3*). During July–August 2020, four state health departments and CDC investigated a COVID-19 outbreak that occurred during a 3-week family gathering of five households in which an adolescent aged 13 years was the index and suspected primary patient; 11 subsequent cases occurred.

Both heads of each household were interviewed to assess demographic characteristics, exposures, symptoms, close contacts, and outcomes. Parents provided data for all children, adolescents, and young adults. Thirteen of the index patient’s relatives sought viral testing; test results were reported by respondents, and all test results that were reported to be positive were verified in state reporting systems. For three children and adolescents who were not tested while symptomatic, a chemiluminescent immunoassay* detecting total antibody to SARS-CoV-2 was performed 28–46 days after symptom onset; results were positive for all three children and adolescents, including the index patient and her two brothers, indicating earlier infection. Likely exposure periods^†^ and infectious periods^§^ were estimated from symptom onset dates. This activity was reviewed by CDC and was conducted consistent with applicable federal law and CDC policy.^¶^

While away from home, the index patient was exposed during a large COVID-19 outbreak in June 2020. Because of her exposure, she sought testing for SARS-CoV-2 after returning home. A rapid antigen test performed 4 days after exposure, when she was asymptomatic, was negative (Table) (Figure). She experienced nasal congestion 2 days later, her only symptom. That same day, she, her parents, and two brothers traveled to a gathering with 15 other relatives, which began the following day. Attendees belonged to five households in four states and ranged in age from 9 to 72 years. Fourteen relatives, including the index patient, stayed in a five-bedroom, two-bathroom house for 8–25 days. These relatives did not wear face masks or practice physical distancing. An additional six relatives (an aunt, an uncle, and four cousins) visited for 10 hours on day 3 and 3 hours on day 10, when six overnight attendees were potentially infectious, but maintained physical distance and remained outdoors; none wore face masks.

**TABLE:** Confirmed, probable, and suspected COVID-19 cases* among overnight attendees and day visitors at a 3-week family gathering — four states, June–July 2020

Patient no.	Relationship to index patient (age, yrs)	Days attended gathering	Symptom onset^†^ (days from start of gathering)	Laboratory testing (results), no. of days from symptom onset	Case status
**Overnight attendee**					
1	Index patient (13)	0–21	−1	Ag (−), −2; Ab (+), 46	Suspected
2	Brother (9)	0–21	2	Ab (+), 43	Suspected
3	Grandfather (72)	0–24	7	PCR (+), 7	Confirmed
4	Mother (42)	0–21	9	PCR (+), 4	Confirmed
5	Uncle (41)	2–5, 7–10	9	Ag (+), 4	Probable
6	Aunt (34)	2–5, 7–10	11	Ag (+), 2	Probable
7	Aunt (46)	0–24	11	PCR (+), 3	Confirmed
8	Uncle (46)	0–12	14	PCR (+), 2	Confirmed
9	Father (42)	0–21	17	PCR (+), 0	Confirmed
10	Grandmother (72)	0–24	17	PCR (+), 3	Confirmed
11	Brother (15)	0–21	17	Ab (+), 28	Probable^§^
12	Cousin (10)	0–24	18	Not tested	Probable^§^
—	Cousin (14)	0–12	N/A	PCR (−), N/A	Noncase
—	Cousin (16)	0–24	N/A	Not tested	Noncase
**Day visitor**					
—	Uncle (48)	3, 10	N/A	PCR (−), N/A	Noncase
—	Aunt (47)	3, 10	N/A	PCR (−), N/A	Noncase
—	Cousin (22)	3, 10	N/A	PCR (−), N/A	Noncase
—	Cousin (20)	3, 10	N/A	Not tested	Noncase
—	Cousin (18)	3, 10	N/A	Not tested	Noncase
—	Cousin (16)	3, 10	N/A	PCR (−), N/A	Noncase

**Abbreviations:** Ab = antibody; Ag = antigen; COVID-19 = coronavirus disease 2019; N/A = not applicable; PCR = polymerase chain reaction.

* Cases were classified as confirmed, probable, or suspected according to revised interim case definitions from the Council of State and Territorial Epidemiologists (https://wwwn.cdc.gov/nndss/conditions/coronavirus-disease-2019-covid-19/case-definition/2020/08/05/).

^†^ Reported signs and symptoms included fatigue (seven patients), measured or subjective fever (six), chills (six), cough (six), loss of smell (five), loss of taste (five), congestion (five), headache (five), shortness of breath (four), myalgia (three), diarrhea (three), runny nose (two), sore throat (one), and nausea (one).

^§^ Based on clinical and epidemiologic criteria.

**FIGURE:**
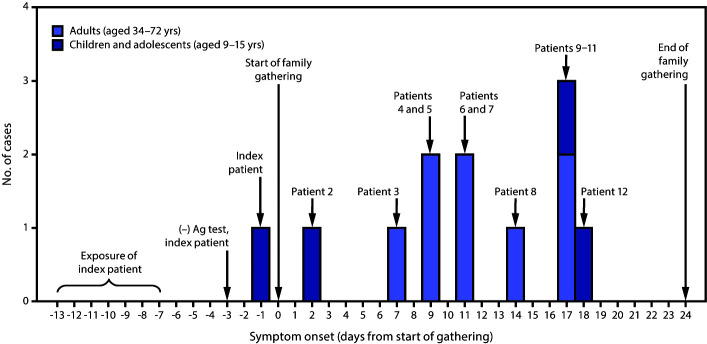
COVID-19 cases among children, adolescents, and adults who attended a 3-week family gathering* — four states, June–July 2020 **Abbreviations:** Ag = antigen; COVID-19 = coronavirus disease 2019. * Patient numbers refer to those in the Table, where further details about each patient are provided.

Among the 14 persons who stayed in the same house, 12 experienced symptoms** and were subsequently found to have COVID-19 based on Council of State and Territorial Epidemiologists definitions.^††^ Six cases were confirmed by reverse transcription–polymerase chain reaction (RT-PCR) testing, four persons were classified as having probable COVID-19 based on positive antigen testing or clinical and epidemiologic criteria, and two persons were classified as having suspected COVID-19 based on positive antibody testing, including the index patient (Table). The other two overnight attendees never experienced symptoms, including one who received a negative SARS-CoV-2 RT-PCR test result 4 days after the last exposure. One person with COVID-19 was hospitalized and another sought emergency department care for respiratory symptoms; both recovered. None of the six relatives who remained outdoors and maintained physical distance developed symptoms; four had negative RT-PCR test results 4 days after the last exposure, and two were not tested. Relatives with COVID-19 were advised by state investigators to self-isolate, and contacts were advised to self-quarantine.

Eight relatives reported activities outside the gathering during their exposure periods that might have increased their risks for exposure. However, only the index patient reported exposure to a person with confirmed COVID-19 or compatible symptoms outside the family. The index patient’s high-risk exposure and symptom onset 3–19 days before that of any other person at the family gathering support the hypothesis that this adolescent’s infection was the source of the family outbreak (Figure). The adolescent’s initial antigen test result was likely a false negative because it was performed before symptom onset; the only antigen test that had Food and Drug Administration Emergency Use Authorization at the time was intended for use within the first 5 days of symptoms.^§§^

This outbreak highlights several important issues. First, children and adolescents can serve as the source for COVID-19 outbreaks within families, even when their symptoms are mild (*2*). Better understanding of transmission by children and adolescents in different settings is needed to refine public health guidance. Second, this investigation provides evidence of the benefit of physical distancing as a mitigation strategy to prevent SARS-CoV-2 transmission. None of the six family members who maintained outdoor physical distance without face masks during two visits to the family gathering developed symptoms; the four who were tested for SARS-CoV-2 had negative test results. Third, rapid antigen tests generally have lower sensitivity (84.0%–97.6%) compared with RT-PCR testing; negative results should be confirmed with RT-PCR if used for persons with high pretest probability of infection, such as those with a known exposure (*4*). Fourth, regardless of negative test results, persons should self-quarantine for 14 days after a known exposure (*5*) or after travel when mandated by state, territorial, tribal, or local authorities (*6*). Finally, SARS-CoV-2 can spread efficiently during gatherings, especially with prolonged, close contact. Physical distancing, face mask use, and hand hygiene reduce transmission; gatherings should be avoided when physical distancing and face mask use are not possible (*7*).

## Footnotes

* https://www.fda.gov/media/136967/download.

† The likely exposure period was estimated to begin 14 days before symptom onset and end 2 days before symptom onset, which corresponds to the longest potential incubation period. https://www.cdc.gov/coronavirus/2019-ncov/hcp/clinical-guidance-management-patients.html.

§ The infectious period was estimated to begin 2 days before symptom onset and end 10 days after symptom onset, according to CDC guidance. https://www.cdc.gov/coronavirus/2019-ncov/php/contact-tracing/contact-tracing-plan/investigating-covid-19-case.html.

¶ See e.g., 45 C.F.R. part 46.102(l)(2), 21 C.F.R. part 56; 42 U.S.C. §241(d); 5 U.S.C. §552a; 44 U.S.C. §3501 et seq.

** Reported signs and symptoms included fatigue (seven patients), measured or subjective fever (six), chills (six), cough (six), loss of smell (five), loss of taste (five), congestion (five), headache (five), shortness of breath (four), myalgia (three), diarrhea (three), runny nose (two), sore throat (one), and nausea (one).

†† https://wwwn.cdc.gov/nndss/conditions/coronavirus-disease-2019-covid-19/case-definition/2020/08/05/.

§§ https://www.fda.gov/media/140299/download.
